# Case in Private Practice

**Published:** 1856-01

**Authors:** Jno. R. Allen

**Affiliations:** Prof. of Obstetrics, &c., College of Physicians and Surgeons, Iowa University


### CASE IN PRIVATE PRACTICE,

REPORTED BY JNO. R. ALLEN, M. D.,

Prof. of Obstetrics, &c., College of Physicians and Surgeons, Iowa University.

About 2 o'clock, P. M., Dec. 23d, I was called to see B——, æt 20 months. This child had been attacked about 11 o'clock, A. M., with great sickness of stomach and active vomiting. The matter discharged was white, glary, and tenacious, mixed with the water taken into the stomach, but not dissolved in it. There had been two dejections from bowels of a fluid not unlike rice water; in quantity near half a gallon.

I found her in a state of great exhaustion; no pulse was to be felt at the wrist; the carotid beat was frequent and feeble; the upper extremities and surface generally were very cold; the skin however, firm and dry, no clamminess; the face was pale and sunken; eyes dull and half closed; great jactitation, and constant effort to uncover the shoulders and arms. The muscular movements were tremulous, with partial loss of their voluntary control. Constant demand for water, which was rejected by the stomach as soon as swallowed.

Death from exhaustion seemed imminent. Ordered hot mustard bath, followed by friction, and sinapisms over whole belly and to extremities. Internally a teaspoonful of brandy with twenty drops Paragoric every hour, with milk and water to allay thirst.

Satisfied that some unusual cause existed for the state of things, enquiry was now made into all the circumstances. We learned that about 10 o'clock, A. M., the child had found a box of Percussion caps, which fell accidentally in its way, and when discovered had some in its mouth. They were immediately taken from it, and, as was supposed, none had been swallowed; two having fallen from its mouth, partially crushed by the teeth.

We were satisfied that some had been swallowed and were the cause of the attack. Ordered demulcents and drinks containing albumen, such as flour and water, white of eggs, flax seed tea, &c., which were allowed as often as drink was demanded, which was, at most, every five minutes.

After the stimulating means had been used for an hour or two, evidences of reaction came on, indicated by some warmth of surface and color of face—no pulse, however, at wrist.

By 9 o'clock P. M., pulse could be slightly felt, very rapid and feeble. There had been no vomiting for several hours. At 2 o'clock A. M., (24th) a pretty copious discharge from bowels again occurred, of same kind of matter, bringing with it *twenty caps* in various stages of corrosion. After this the circulation gradually came up, (stimulants discontinued) going ultimately a little above natural standard. The nervous system was greatly disturbed, the child started from its short periods of repose and screamed, manifesting much alarm and muscular tremor. The thirst was still very urgent, the lips and tongue of a florid red and perfectly dry.

Laudanum in three drop doses every two hours, was ordered until sleep was induced, still allowing the drinks which were taken cold. We apprehended active gastro-interitis, but happily a high state of irritation only was induced, and in two days more, the little patient was on its feet.

This case is interesting, as exhibiting the results of great mechanical irritation, as well as some of the poisonous effects of the copper. I do not know the composition of the detonating powder in the caps, but we do know that it is sometimes made of ingredients which would, in sufficient quantity, be poisonous.

Cases might easily occur and indeed have happened, where fatal results have followed the introduction of a single cap into the prima via; where it has lodged in some of the valves of the canal, acting as a focus of irritation, both by its mechanical action and the irritant effects of its poisonous qualities. Here, however, we have a very nervous child, who has taken 20 into its digestive tube and resulting as reported—but another evidence of the unnumbered and unknown modifying agencies which affect the causes and course of disease.

